# Practical aspects of the diagnosis and management of pyoderma gangrenosum

**DOI:** 10.3389/fmed.2023.1134939

**Published:** 2023-02-14

**Authors:** Bo Chen, Wei Li, Bin Qu

**Affiliations:** Department of Burns, Sichuan Academy of Medical Sciences and Sichuan People’s Hospital, Chengdu, China

**Keywords:** pyoderma gangrenosum, ulcer, surgery, wound healing, chronic wounds

## Abstract

Pyoderma gangrenosum (PG) is a rare autoinflammatory ulcerative neutrophilic skin disease. Its clinical presentation is a rapidly progressing painful skin ulcer with ill-defined borders and surrounding erythema. The pathogenesis of PG is complex and not fully understood. Clinically, patients with PG often have various systemic diseases, the most common being inflammatory bowel disease (IBD) and arthritis. Due to the lack of specific biological markers, diagnosing PG remains difficult, which easily resulting in misdiagnosis. Some validated diagnostic criteria have been applied in clinical practice that facilitate its diagnosis. The treatment of PG currently consists mainly of immunosuppressive and immunomodulatory agents, especially biological agents, which have bright prospects for PG therapy. After the systemic inflammatory response is controlled, the problem of wounds becomes the main contradiction in PG treatment. Surgery is not controversial for PG, increasing evidence shows that with adequate systemic treatment, the benefits of reconstructive surgery for patients are increasing.

## 1. Introduction

Pyoderma gangrenosum (PG) is a rare autoinflammatory ulcerative neutrophilic skin disease that was first described by Brocq in 1908. The term pyoderma gangrenosum, suggested in 1930 by Brunsting, was ultimately adopted (1). PG was originally considered related to occult bacterial infection, autoantibodies in the blood, and a phenomenon called the Shwartzman reaction (2, 3); however, subsequent studies have shown no clear evidence to support these theories. PG is currently classified as a neutrophilic skin disease, mainly because of its pathological manifestations of massive accumulation of neutrophils in the skin and subcutaneous tissue. However, the cause of the inflammatory process of PG remains unclear (4). In fact, the name pyoderma gangrenosum is not appropriate, as the opposite is true. It is neither an infectious disease nor a gangrenous disease, and it currently tends to be attributed to an autoimmune disease caused by an abnormal immune response, neutrophil dysfunction (4, 5), genetic alterations (e.g., MEFV and PSTPIP1 mutation) (6–8), and dysregulation of the innate immune system (9, 10), which are now considered the main reasons for its development.

This review summarizes the new progress in the diagnosis, medical treatment, and surgical treatment of PG to help wound repair physicians better understand and manage this disease and ultimately improve its standardized diagnosis, treatment and prognosis.

## 2. Clinical features

### 2.1. Epidemiology

At present, there is little epidemiological data on PG, and the literature shows that its incidence, age at onset, and sex preference differ, likely due to diagnostic difficulty and the lack of a gold diagnostic standard recognized by consensus. However, an analysis of the literature revealed that the incidence of PG is relatively low, the age distribution is wide [including infants, which account for approximately 4% of cases of PG and the elderly (11, 12)], and it has also been reported in pregnant women (13, 14). Statistics published by British scholars showed that the incidence was 0.63/100,000 individuals, the sex difference was not significant (male vs. female = 41% vs. 59%), and the median age at onset was 59 years (15). According to studies published by American scholars, the incidence of PG is 5.8–20/100,000, and there is a significant sex difference. There are approximately twice as many females as male patients (male vs. female = 32–37% vs. 63–68%). Patients over 50 years of age account for nearly 70% of all PG cases (16, 17). The epidemiological data from Asia published by Japanese scholars may be representative. The reported incidence was 0.3/100,000, the sex difference was not significant (male vs. female = 44% vs. 56%), and the proportion of patients over 50 years of age was approximately 65% (18). Regarding its etiology, a retrospective analysis of small cases published by Schosler et al. (19) showed that 49% of patients developed the disease spontaneously, 27% developed it after minor trauma, and 17% developed it after undergoing major surgery or tissue therapy.

### 2.2. Comorbidities

Approximately 50% of PG patients have systemic diseases, the most common being inflammatory bowel disease (IBD), arthritis, solid organ malignancies, and hematological malignancies. The proportion of specific comorbid diseases varied among different studies (15, 20, 21). Other diseases associated with PG, such as hepatitis C, have also been rarely reported (22). Although PG is highly associated with these diseases, the risk of its development among patients with highly related diseases is relatively low (23). This suggests that PG cannot be considered a separate disease and should be viewed systematically with careful assessment for the existence of related systemic diseases in affected patients. In PG-related diseases, the precipitating factors should be treated with caution, and patients alerted to its complications. There are also reports in the literature that drugs used to treat PG may paradoxically lead to its occurrence (24), which brings confusion and illustrates its complexity.

#### 2.3. Clinical presentation

The clinical manifestations of PG vary among studies. The classic type is ulcerative PG, which is very painful, usually begin as papulopustules (or blisters or nodules), and then undergo necrosis leading to ulcer formation. Lesion development is often rapid and the level of pain is usually greater than expected based on the appearance of the ulcer. Pathologic changes appear as moth-eaten ulcerations, often involving the subcutaneous fat layer. The skin at the edge of the ulcers is cherry or purplish red, and the underlying healthy tissue becomes destroyed. The clinical classification of PG and related systemic diseases is presented in Table 1 (23, 25, 26). This information suggests that the most common type of PG is the ulcerative type, and the most common site is the outside of the lower leg, which may be related to the exposure of this site and greater chance of injury. In addition, reports of postoperative PG and PG in special parts of the body are increasing and should be considered. The scars remaining after PG lesion healing usually have a cribriform or puckered appearance.

**TABLE 1:** Clinical variants of PG (23, 25, 26).

Variant	Clinical presentation	Common locations	Histopathology	Reported associated systemic diseases
Ulcerative (Classic form)	Tender inflammatory nodules or pustules that rapidly evolve into necrotic ulcers with violaceous undermined borders and surrounding erythema	Most commonly occurs at sites of trauma, frequently on the anterior lower extremities	Findings depend on the location and stage of the lesions. Biopsy samples taken from the ulcer edge show neutrophils and perivascular lymphocytic infiltrates with dermal oedema, whereas biopsy samples taken from the center show a predominantly neutrophilic infiltrate. Vascular damage with fibrin deposition, thrombosis and red blood cell extravasation is common.	IBD, hematological malignancies, rheumatoid arthritis, seronegative arthritis and monoclonal gammopathy
Bullous	Rapidly evolving, painful bulla(e) that can progress to erosion and/or ulceration	Face, upper extremities more often than lower extremities	Subcorneal, subepidermal and intraepidermal bullae with dermal neutrophilic infiltrate and microabscess formation. Immunofluorescence is negative or non-specific, which helps to rule out immunobullous diseases.	Myeloproliferative disorders (especially acute myeloid leukaemia) and IBD
Pustular	Pustules with symmetric erythematous borders	Legs and trunk	Neutrophilic infiltrate and accumulation underneath the stratum corneum (subcorneal), around hair follicles and in the derma, with subepidermal oedema	IBD
Vegetative	Less-painful variant, slow-growing, non-purulent, often a single superficial ulcer; borders are not undermined and less violaceous; readily responsive to therapy	Trunk	Palisading granulomatous reaction (mononuclear cells with elongated or spindle-shaped nuclei palisaded around the edge of the central necrotic zone) and neutrophilic abscesses with sinus tracts.	None
Peristomal	Papules that erode into ulcers with undermined borders; often difficult to distinguish from other peristomal erosive lesions	Immediately adjacent to the stoma	Dermal neutrophilic infiltrates with granulation tissue	IBD, enteric malignancy, connective tissue disease and monoclonal gammopathy
Postoperative	Erythema at the surgical site, followed by wound dehiscence or ulcerations that coalesce. Pain out of proportion to examination expectations.	At the surgical site	Dermal oedema and neutrophilic infiltrate	Commonly associated with abdominal and breast surgery

## 3. Diagnostic criteria

Because the clinical, histopathological, and laboratory examinations of PG are non-specific, and recognized and validated diagnostic criteria for PG are currently lacking, it is prone to misdiagnosis. Therefore, when a PG lesion is suspected, a comprehensive evaluation should be performed based on the patient’s medical history. Although it has been reported in the literature that PG onset is inconsistent with the status of related diseases (1, 2), we still believe that a history of previous diseases is important in PG patients. Because PG has also been reported after the use of certain drugs (24, 27–29) and the abovementioned PG-related diseases have often been found in such patients when the past medical history is investigated, the relationship between drugs and PG may require further research.

Diagnostic criteria published by Su et al. (30) in 2004 include two major criteria and two minor criteria and requires the exclusion of other diagnoses, which is very difficult in clinical practice. In 2018, the diagnostic criteria for ulcerative PG developed by the Delphi International Expert Consensus (31) were more useful than those developed by Su et al. (30) in clinical practice. The criteria covered histology, medical history, clinical examination, and treatment response. In 2019, Jockenhofer et al. (32) proposed diagnostic criteria for PARACELSUS, which used a score-based approach in which the weight of each criterion was determined by the prevalence observed in PG patients. These two diagnostic criteria are improvements to and simplifications of the diagnostic criteria proposed by Su et al. (30). When diagnosing PG, researchers try to weaken the importance of excluding other suspicious diseases that cause ulcers and focus more on the pathological features of PG. A histological examination ideally renders a definitive diagnosis; however, this does not reflect clinical reality (33). In a cross-sectional retrospective study (34) evaluating three diagnostic criteria, the authors concluded that the PARACELSUS score had the highest proportion of confirmed diagnoses (approximately 90%). Another multicenter evaluation of the diagnostic criteria for ulcerative PG indicated that the PARACELSUS might outperform Delphi in a real-world setting (35, 36). However, it is undeniable that there are many confounding subjective factors, such as medical history and patient experience, in these three diagnostic criteria that may lead to misleading diagnoses by medical staff. Moreover, they all pay more attention to the diagnosis in the acute stage. For chronic ulcers, the pathological manifestations may be atypical and coinfection may occur. The diagnostic criteria pay little attention to this aspect. Table 2 (30–32) compares the three diagnostic criteria.

**TABLE 2:** Comparison of the different diagnostic criteria suggested for PG (30–32).

	Su et al. criteria^a^	The Delphi Consensus of International experts^b^	PARACELSUS Score^c^
Major criteria	Rapid progression of a painful, necrolytic cutaneous ulcer with an irregular, violaceous, and undermined border	Biopsy with a neutrophilic infiltrate	Progressing disease (3 points)
	Exclusion of other causes of cutaneous ulceration		Assessment of differential diagnoses (3 points)
			Reddish-violaceous wound border (3 points)
Minor criteria	History suggestive of pathergy or clinical finding of cribriform scarring	Exclusion of infection on histology	Amelioration by immunosuppressant drugs (2 points)
	Systemic diseases associated with PG	Pathergy	Characteristically irregular (bizarre) ulcer shape (2 points)
	Histopathologic findings (sterile dermal neutrophilia, ± mixed inflammation, ± lymphocytic vasculitis)	Personal history of IBD or inflammatory arthritis	Extreme pain > 4/10 on visual analog scale (2 points)
	Treatment response (rapid response to systemic steroid treatment)	Papule, pustule, or vesicle that rapidly ulcerates	Localization of lesion at site of trauma (2 points)
		Peripheral erythema, undermining border, and tenderness at the site of ulceration	Suppurative inflammation in histopathology (1 point)
		Multiple ulcerations (at least one occurring on an anterior lower leg)	Undermined wound border (1 point)
		Cribriform or wrinkled paper scars at healed ulcer sites	Systemic disease associated (1 point)
		Decrease in ulcer size after immunosuppressive treatment	

^a^Diagnosis requires both major criteria and at least two minor criteria.

^b^Diagnosis requires meeting the major criterion and at least four of eight minor criteria.

^c^Points ≥ 10, PG highly likely; points < 10, PG unlikely.

As there is no definitive test for diagnosing PG, clinical misdiagnosis often occurs. Weenig et al. (37) reported a misdiagnosis rate of approximately 10%. However, owing to referral bias, the actual misdiagnosis rate may be much higher. Disorders that require differentiation from PG include necrotizing fasciitis, antiphospholipid antibody syndrome, vaso-occlusive disease, venous disease, vasculitis, malignancy, skin infection, tissue damage induced by drugs or trauma, and ulcerative inflammatory disease (37–41). Based on the above characteristics of PG, we recommend the related tests listed in Table 3 (20, 26, 37, 42). Since PG involvement of extracutaneous organs has also been reported (43), attention should be given to PG skin ulcers and comorbidities, with careful assessment for evidence of other organ damage.

**TABLE 3:** Recommendations for the laboratory tests (20, 26, 37, 42).

Skin biopsy	Include an inflamed border and ulcer edge at a depth that includes the subcutaneous fat
Chest radiography	To evaluate for possible infection prior to the initiation of immunosuppressive therapy
Colonoscopy	To evaluate for underlying inflammatory bowel disease
Tissue culture	To detect bacteria, fungi, and atypical mycobacteria
Doppler or angiography	Venous and arterial function studies
Complete blood count	To evaluate for underlying hematologic disorders
Blood chemistry	Liver- and kidney-function tests
Antineutrophil cytoplasmic antibodies (ANCA)	To evaluate for granulomatous vasculitis
Rheumatoid factor	As a component of the evaluation for cryoglobulinemia and rheumatoid arthritis
Antinuclear antibody titre	To evaluate for systemic lupus erythematosus or collagen vascular disorders
Antiphospholipid antibody	To evaluate for the presence of antiphospholipid syndrome
Hepatitis testing	To evaluate for associated hepatitis B or C, particularly for patients in whom immunomodulatory therapy is considered

## 4. Treatment progress

Before treatment begins, we re-emphasize that careful exclusion of other possible causes of skin ulceration should be the first and most important step in the management of PG because its treatment requires the use of immunosuppressive drugs, which are usually contraindicated for most other causes of skin ulcers.

Although there are many reported cases of PG treatment, most are expert recommendations, case reports or retrospective analyses with small sample sizes. We retrieved only two published randomized trials (44, 45), and such studies are lacking. As a result, many clinical questions remain unanswered. However, suggestions for the management of PG are provided by the current clinical and basic research evidence, which has been recently reviewed by Maverakis et al. (23). First, other diseases that may cause skin ulcers must be ruled out. Second, PG is an immune-mediated disease; therefore, it is necessary to use immunosuppressive or immunomodulatory drugs in a timely manner to prevent the progression of abnormal inflammation. Third, PG wounds require professional wound treatment, and surgical reconstruction may be necessary when the circumstances permit them. Fourth, the treatment of coexisting diseases should also be considered when choosing a treatment strategy. Although PG symptoms do not parallel the manifestations of coexisting diseases, the treatment of coexisting diseases sometimes improves them (26, 46). Fifth, improper pain treatment may lead to the occurrence of stress, anxiety, and depression (47), negatively affecting patient quality of life and delaying or inhibiting wound healing. Topical drugs, non-steroidal anti-inflammatory drugs, or opioids can be used to relieve pain.

### 4.1. Topical therapy

Topical therapy is often the initial treatment of choice for patients with mild localized lesions (< 2 cm^2^). Corticosteroids and/or calcineurin inhibitors are often used for ulcers and abnormal areas around them. Complete healing usually takes a long time, ranging from several weeks to several months. Other topical treatments included sodium chromate, nicotine, dapsone, and 5-aminosalicylic acid (26, 48). There are no randomized trials of topical or intra-ulcer wound treatment, and medication type, dose, and frequency remain unstandardized (49).

### 4.2. Systemic treatment

Since the first report in 1956 by some scholars of the successful treatment of PG with systemic corticosteroids (50), the immunological treatment of PG has received widespread attention and, subsequently became the first-line treatment. Because PG is a rapidly progressive disease, the use of fast-acting immunosuppressants such as cyclosporine or corticosteroids is usually more effective (45). A large randomized controlled trial (51) showed that cyclosporine and prednisolone were associated with similar rates of wound healing (47 vs. 47%), ulcer recurrence (30 vs. 28%), and adverse reactions (68 vs. 68%). There was a significant difference in the rate of serious adverse reactions (3 vs. 13%); serious infections accounted for the majority of serious adverse reactions (approximately 70%). During corticosteroid treatment (26), oral prednisone can be selected at a dose of 0.5–1.0 mg/kg/d, and the maximum dose does not exceed 60 mg/d or other equivalent doses. For rapidly progressing PG, pulse therapy can also be used with intravenous methylprednisolone 1 g/d for 1–5 days. The usual dose of cyclosporine is 2.5–5.0 mg/kg/d (26).

However, it has been reported that the effective rate of parenteral administration of systemic corticosteroids is significantly higher than that of oral therapy (52), suggesting that in the rapidly progressive stage of PG, intravenous therapy may be preferred, while oral therapy may be more appropriate once the disease has stabilized or the patient has been transferred to an outpatient clinic. The systemic use of corticosteroids usually has a rapid onset of action, and the condition stabilizes within 2–3 days (53). The patient’s direct experience is pain improvement as it takes significant time for the ulcer to heal completely. The long-term use of corticosteroids is associated with serious adverse reactions. Therefore, after observing the improvement of clinical indicators (stop in ulcer progression and reduction in pain, inflammation, and wound area), which is referred to as Gulliver’s sign (54), the dosage of corticosteroids should be reduced in a timely manner. The dose reduction should be carefully implemented, and it is inappropriate to reduce it too much at once or stop it suddenly. The use of other immunosuppressive agents such as methotrexate, aminophenolic acid, azathioprine, and sulfasalazine for the treatment of PG has also been reported (26). Although some therapeutic effects have been achieved, objective indicators to verify the therapeutic effects are still lacking (55).

### 4.3. Biologics

Targeted therapy has changed the treatment of many diseases, including PG, and biologics have shown great potential as second-line therapies. The major biologics include anti-tumor necrosis factor drugs (e.g., infliximab, adalimumab, etanercept, golimumab and certolizumab), anti-interleukin (IL)-12/IL-23 drugs (e.g., ustekinumab), anti-IL-23 drugs (e.g., tildrakizumab), a receptor antagonist (e.g., anakinra); IL-1ß antagonists (e.g., canakinumab and gevokizumab), anti-IL-6 receptors (e.g., tocilizumab), anti-IL-17 drugs (e.g., secukinumab), and JAK-STAT inhibitors (e.g., tofacitinib and ruxolitinib) (23, 25, 26, 56). In 2006, Brooklyn et al. (44) evaluated the efficacy of infliximab versus placebo before publishing the first randomized controlled trial on PG, demonstrating the efficacy of infliximab. Other reports of biologic therapy are mostly case reports or small cases, and there are also instances of biologics causing “paradoxical reactions”, that is, phenomena that occur or worsen during the use of biologics, often as a result of these drugs (57).

### 4.4. Intravenous immunoglobulin

Intravenous immunoglobulin (IVIG) is often used to treat patients with severe PG who are refractory to first- and second-line drugs, with a treatment efficacy rate of approximately 88% (58–60). These patients received IVIG with systemic corticosteroids, suggesting that IVIG may be an effective adjunct therapy for refractory PG. However, considering the cost of IVIG treatment and adverse reactions, such as fever, allergic reaction, headache, nausea, and aseptic meningitis (58), prospective clinical trials are needed to evaluate and verify its efficacy.

### 4.5. Wound treatment

Wound treatment is an important component of PG treatment. The goal of wound treatment is to create an optimal healing environment. The treatment strategy for PG wounds should use reasonable means to treat wounds controlled by immunosuppressive agents. Factors affecting other types of wounds, such as infection, bacterial colonization, and systemic conditions, also affect PG wound healing (23).

The wound should be cleaned with lukewarm sterile saline or mild disinfectant to reduce irritation and relieve pain. The choice of dressing should be based on the amount and shape of wound exudate. It may be appropriate to choose an absorbent antibacterial dressing when the amount of exudate is large in the early stage. When wound exudation decreases, more attention should be paid to the antibacterial properties of the dressing to reduce bacterial colonization within the wound. In the later stage, the amount of wound exudation continues to decrease and crusts form. Wet antibacterial dressings are used to maintain appropriate humidity of the wound to facilitate wound healing. In conclusion, the dressing strategy should be tailored to the wound’s characteristics. We found that antimicrobial dressings and hyper absorbent dressings were the most appropriate for managing PG wounds. Modern dressings require less frequent changes and manipulation (61).

Although PG wounds are initially sterile, the use of antibiotics is generally not recommended; however, bacterial colonization and infection may occur during treatment. This complication may be related to immunosuppressive therapy, delayed diagnosis, or skin barrier dysfunction (37). When PG is indistinguishable from infectious skin and soft tissue diseases, tissue culture should be performed to provide a differential diagnosis. However, culture results often take a long time, which may delay disease management. The rapid molecular diagnosis of pathogens, such as next-generation sequencing, is useful for infectious diseases (62, 63). When wounds caused by deep fungal, leishmanial, or mycobacterial infections are difficult to distinguish from PG wounds, the rapid molecular detection of pathogens has more advantages than traditional detection methods. When wounds become chronic, infection or bacterial colonization may occur; when sensitive antibiotics are used and the wound condition does not obviously improve, the accuracy of the diagnosis requires re-evaluation.

## 5. Advances in surgical treatment

For wounds caused by other factors, surgical treatment is an important step. The wounds of PG patients exhibit pathergy; that is, PG occurs in the skin of accidental or iatrogenic wounds, or the original PG lesion deteriorates. The probability of PG occurrence in patients after surgical procedures varies among studies (15–20%) (64–67). Owing to the lack of specific biomarkers for PG, it is not realistic to predict the occurrence of PG. Therefore, other phenomena that lead to PG in the surgical area after other operations are often reported (68, 69), and the disease is easily misdiagnosed as necrotizing fasciitis or other serious infections in the early stages (70), thereby misguiding the surgeon to conduct more active debridement and leading to serious consequences (71). Haag et al. conducted a literature review of perioperative management practices and risk factors that may predict the response to surgical intervention (67).

However, if PG wounds have a large wound area, high risk of infection, or exposure of important tissue structures, although immunosuppressive therapy can slow or prevent PG progression, it cannot solve the problem of wound prevention, the main contradiction of PG. The optimal timing of surgical treatment is currently unknown; however, we believe that with adequate systemic treatment, the inflammatory response stops, the condition stabilizes, and surgery may be beneficial. There are many options for surgical reconstruction depending on defect extent and location, including autologous skin grafts, allogeneic skin grafts, xenograft skin grafts, negative pressure wound therapy (NPWT), and free flap grafts (72–75). The positive therapeutic effect of NPWT in other wounds has been confirmed. For PG wounds, when the systemic inflammatory response is well controlled, NPWT can reduce the wound area, control wound exudation, reduce dressing changes and possibly relieve pain (76). Local flap surgery is generally not recommended because of the possibility of local progression of wounds and pathergy. Under sufficient systemic treatment, it may be better to use simple surgical methods to seal PG wounds as soon as possible, such as autologous skin combined with NPWT, which can reduce the number of dressing changes after surgery and reduce PG wound irritation.

Unfortunately, no specific measures have been identified to prevent the initial occurrence of PG. For patients diagnosed with PG, avoiding trauma can reduce the occurrence of new wounds (66). In addition to related systemic diseases, possible high-risk factors include obesity, female sex, and certain drugs (mostly colony-stimulating factors and small-molecule tyrosine kinase inhibitors) (26, 77, 78).

## 6. Conclusion

PG has a long history of use. Although various discoveries have been made regarding its pathogenesis, none have clearly explained its pathogenesis. Most PG studies to date are retrospective clinical analyses, such as expert reviews, case reports, and case summaries, and their quality is generally not high. Only two randomized controlled trials of PG have been published. This may be due to inconsistent diagnostic criteria, different uses of diagnostic tools, low morbidity, and a low number of cases. In the future, we should continue to conduct in-depth research on PG pathogenesis, clarify its molecular mechanism, and discover specific biomarkers to guide its diagnosis, severity assessment, treatment effect evaluation, and early warning of recurrence. More clinical randomized controlled trials are needed to explore the optimal doses and combinations of immunosuppressive and immunomodulatory drugs used alone or in combination with existing treatments.

## Author contributions

BC, WL, and BQ: conceptualization. BQ: methodology, supervision, and project administration. BC: formal analysis, investigation, data curation, writing—original draft preparation, and visualization. WL and BQ: writing—review and editing. All authors have read and agreed to the published version of the manuscript.
